# Improving care seeking behavior toward cervical cancer screening participation among Gwafan community women, North-Central Nigeria

**DOI:** 10.1186/s12905-023-02353-9

**Published:** 2023-07-04

**Authors:** Eunice Samuel Ari., Regidor III Poblete Dioso, John Obafemi Sotunsa

**Affiliations:** 1 Faculty of Nursing, Lincoln University College, Kuala Lumpur, Malaysia; 2 Benjamin S. Carson (Snr) College of Health and Medical Sciences, Babcock University, Ilishan Remo, Ogun State Nigeria

**Keywords:** Care-seeking behavior, Cervical cancer, Human center design, Screening participation

## Abstract

**Introduction:**

Cervical cancer is the fourth most common cancer in women globally and the second most common cancer in low- to middle-income countries, and its screening rate is yet to reach the 70% WHO target. Most interventions that proved effective in improving screening participation in some communities did not achieve the desired behavioral outcome in some settings.

**Aim:**

This study aimed to evaluate the effectiveness of care-seeking behavior interventions on cervical cancer screening participation.

**Method:**

A pragmatic multiphase mixed methods design was adopted for this study, and three phases of the human-centered design process were used for data collection. Deductive thematic analysis was used for qualitative data, while SPSS was used for quantitative data analysis.

**Results:**

The findings show a significant relationship between participants’ tribes p values (0.03) 0.05 and screening participation. Before the intervention, most (77.4%) were afraid of exposing their private parts; 75.9% were afraid of being diagnosed with cervical cancer; and the majority felt the procedure was embarrassing and painful. Free screening, awareness, and knowledge, offering transport, the use of influencers, and sample collection by a female care provider are among other facilitators to screening. Screening participation improved from 11.2% preintervention to 29.7% postintervention (average mean screening score from 1.890.316 to 1.70000.458). All participants who were screened postintervention said the procedure was not embarrassing or painful and that they were not afraid of the procedure or the screening environment.

**Conclusion:**

In conclusion, screening habits in the community were low before intervention, as this may have resulted from women’s feelings and past experiences with screening services. Sociodemographic variables may not directly predict screening participation. Care-seeking behavior interventions have significantly increased screening participation postintervention.

## Introduction

Cervical cancer (CC) is caused by a sexually acquired infection with the human papillomavirus (HPV). Globally, this is the fourth most common cancer in women, with an estimated 604 127 new cases and 341 831 deaths in 2020 [1]. At the regional level, cervical cancer is the second most common in Africa, with 117 316 new cases and 117 316 deaths, with rates seven to ten times higher than in Australia, Western Asia, North America, and New Zealand [1]. In low- and middle-income countries, CC is the second most common cancer and the major cause of death among women. It is the second most common cancer and the leading cause of cancer-related deaths in Sub-Saharan Africa [1]. There are 50.33 million Nigerian women aged 15 and older who are at risk of developing cervical cancer, 14,943 of whom are diagnosed, and 10,403 of whom die yearly [2]. The worrisome rise in adolescent and young adult patients with malignant epithelial malignancies of the cervix is alarming among patients aged 0–30 years, especially the 21–30 year age group [3].

Cervical cancer is preventable worldwide through screening, detection, and treatment of precancerous abnormalities [4] in order to prevent the cervical dysplasia from transforming to cervical cancer [5]. Cervical cancer screening guidelines recommend that women between the ages of 21 and 29 should receive a Pap test every three years; women between the ages of 30 and 65 should receive an HPV test every five years or a Pap test every three years after consulting with their doctor; and those over the age of 66 should ask their doctors if they need continued screening testing every five years [6]. According to projections, the burden of the disease will rise to nearly 460,000 deaths by 2040 if preventive services are not scaled up quickly [7]. Cervical cancer screening rates have declined in Western countries and among women between ages 45 and 65 in the United States [8]. Screening uptake is declining in low-resource countries [9]. In Nigeria, there is a paucity of information on the screening rate, but some studies conducted in parts of the country found that cervical cancer screening coverage by conventional cytology was less than 9% in the populace [10], 10.2% among federal civil servants in north central Nigeria [11], and 32.6% among health workers at JUTH and its environs [12]. Most cervical cancer cases (72.3%) are diagnosed at advanced stages [13]. Women use cervical cancer screening services at a relatively late age, with a median age of 37 [14], and 40–49 years [15]. Fear and embarrassment [16], [17], [18] [19], [20], [21], [22]low socioeconomic status [6], and unsatisfactory previous screening experiences [23], [24], may all contribute to low uptake.

Interventions which were effective in improving screening in some settings did not achieve their desired aim in other settings as found in these studies: Free screening did not increase screening rate [8], Availability of screening site near women homes (5 km away from their homes) did translate to screening [22], Educational interventions were not very effective overall [25], [26] Economic incentivization interventions were moderately effective, increased uptake but still achieving less than 20% coverage [27] very high willingness to screen, even preintervention, but that intent was not always well-translated to the uptake of cervical cancer screening [28], increase from 75.8 to 91.0% in willingness to screen yet no change in actual screening [26], significant improvement in uptake without any community health educators [29], pre-invitation leaflet nor online booking did not increase uptake by three months (18.8% pre-invitation leaflet vs. 19.2% control and 17.8% online booking vs. 17.2% control. The offer of a nurse navigator, a self-sample kit on request, and a choice between timed appointments and a nurse navigator were ineffective [30]. In the absence of facilitating conditions, barriers to screening ensued.

To reduce the burden of cervical cancer, women must take up cervical screening services that will detect the presence of HPV and prevent precancerous cells from transforming into cancerous cells, especially in countries where human papillomavirus vaccines are not readily available and accessible. Effective CC screening interventions based on women’s needs are a critical component of prevention that will improve screening participation and eventually reduce the global burden of cervical cancer.

## Materials and methods

A pragmatic multiphase mixed methods design was adopted for this study using three phases of the Human-Centered Design process with the assumption that collecting multiple types of data provides a more comprehensive understanding of a research problem than either quantitative or qualitative data alone and that providing interventions based on women’s needs may improve screening participation. This study began with an inspiration phase and quantitative data collection on the affect and habits of community women regarding screening participation. In the ideation phase, research team discussed and brainstormed on potential interventions that may improve participation in screening, such as free screening, health education, and community outreach, using focused group discussion. An open-ended question was asked to elicit opinions regarding what should be done to improve screening participation. Experts’ opinions were also sought at this phase. Lastly, the implementation phase focused on quantitative data collection to evaluate the implemented interventions on the affects and screening habits of women regarding cervical cancer screening. Each phase of the study was unique and designed to answer a specific question being asked. Pre-intervention data was collected in September 2019, while the post-intervention evaluation was done within 12 weeks after the intervention. Researchers went to clinics for postintervention data from 8 a.m. to 12 p.m. every Monday to Thursday during the period of data collection. The study was conducted in Gwafan community, one of the 22 villages of Gwong District in Jos North Local Government Area (LGA) of Plateau State. It is located about 6 kilometers east of the Jos municipal area, the capital of the state. The community’s population is continuously rising due to the relocation of the Jos University Teaching Hospital (JUTH) to the ward. Its geographical coordinates are 9° 54’ 0” north and 8° 58’ 0” east. Many ethnic groups reside in the area. Most natives residing in this community are Afizere. There is low cervical cancer screening uptake among women in this community, as found among health workers in JUTH and its environs (32.6%) and less than 9% in the populace [9]. The screening for cervical cancer in this study was done at the cervical screening site at JUTH in Jos, Plateau State, Nigeria, which is about 4 to 5 km from the community.

Sample size was determined using a proportion formula $\frac{\left[{z}^{2}\left(p\times q\right)\right]}{{e}^{2}}$. Cochran (1963)

Where:

n = sample size to be calculated.

Z = Confidence level 95% -> Z = 1.96.

e = degree of freedom - (e.g., 5%) = 0.05.

p = 32.6%= 0.326 proportion of women who had ever screened among health workers in JUTH and its neighborhoods [12].

q = 1-p, 1-0.326 = 0.674 n ≈ 340.

Women were selected purposefully. Criteria for inclusion: women aged 21–65 who are sexually active, reside in the Gwafan community, and are willing to participate. Criteria for exclusion: Include participants who were unwilling to discuss or are distressed by discussing cervical cancer; are critically ill; are mentally unstable; are women who had hysterectomy involving removal of the cervix; or were diagnosed with cancer of the cervix. Women were administered questionnaires conveniently on a face-to-face basis. Research instrument was rated: Poverty level: Extremely poor < 284,700.00 Naira, moderately poor = 284,700.00-479,500.00 Naira, and not poor > 479,500 Naira as annual income [31], [32]. Age range: Younger women= [21–29] and older women= (30–65) years. Permission to conduct research in the Gwafan community was sought through the Gatekeeper. Recruitment of eligible candidates was done until the sample size was reached after a leaflet containing details of the study and the researcher’s contact information was given to participants. Quantitative data was collected using a questionnaire consisting of preintervention affect and habits in the inspiration phase and postintervention affect and habits in the implementation phase. A focus group discussion was conducted with four participants in Hausa in the ideation phase, where open-ended questions were read to participants for their responses to help develop interventions that will improve screening participation. The verbatim transcription was done on the same day by the interviewer. All information was collected on a face-to-face basis after written consent and permission for the audio record were obtained and participants were briefed on the aim of the study. Experts’ opinions on how to improve screening participation were documented in the ideation phase. Two female research assistants who were familiar with the community and data collection were recruited.

## Result

### Phase one (inspirational phase)

This phase used a quantitative approach to collect data on socioeconomic variables, affect and habits regarding care-seeking behaviour for cervical cancer screening participation.

Table 1. shows that the majority (52.1%) felt the procedure for cervical cancer screening was embarrassing, 53.2% felt the procedure was painful, 77.4% said fear of exposing their private part, 64.7% said they were afraid of cervical cancer screening procedures and the environment, and 75.9% said they were afraid of being diagnosed with cervical cancer.

**Table 1:** Preintervention Affect of Gwafan community women care-seeking behaviour (September 2021)

Item		Frequency (f)	Percent (%)
**The procedure is embarrassing**	Yes	177	52.1
	No	163	47.9
	Total	340	100.0
**Procedure is painful**	Yes	181	53.2
	No	159	46.8
	Total	340	100.0
**Fear of exposing my private part**	Yes	263	77.4
	No	77	22.6
	Total	340	100.0
**Fear of the procedure and screening environment**	Yes	220	64.7
	No	120	35.3
	Total	340	100.0
**Fear of being diagnosed with the disease**	Yes	258	75.9
	No	82	24.1
	Total	340	100.0

Table 2. shows that the majority (88.8%) of the women had never been screened for cervical cancer, while only 11.2% had been screened for cervical cancer. Out of the 38 women who had screened, 86.8% had a good experience during screening, while 5 (13.2%) had bad experiences.

**Table 2:** Preintervention Habits of Gwafan community women care-seeking behaviour (September 2021)

Item		Frequency (f)	Percent (%)
**I have ever screened for** **cervical cancer**	Yes	38	11.2
	No	302	88.8
	Total	340	100.0
**Year Screened**	< 2018	13	34.2
	2018–2021	25	65.8
	Total	38	100.0
**Experience during previous** **Screening**	Bad procedural experience	5	13.2
	Good experience	33	86.8
	Total	38	100.0

Table 3 shows no significant relationship between age p(0.391) > 0.05; marital status p(0.460) > 0.05; religion p(0.999) > 0.05; number of sex partner p(1.000) > 0.05; occupation p(0.729) > 0.05 and annual income p(0.770) > 0.05 and screening participation. There is, however, a significant relationship between tribe p(0.030) < 0.05 and the Uptake of cervical cancer screening.

**Table 3:** Binary logistic regression of socio-demographic factors (Tribe, Marital status, Religion, Number of sex partners, Family type, Occupation and Annual income. (n = 340)). (September 2021)

		B	S.E.	Wald	df	Sig.	Exp(B)	95% C.I.for EXP(B)	
								Lower	Upper
	**Age**	1.040	1.213	0.735	1	0.391	2.829	0.262	30.490
	**Tribe**								
	Afizere			6.987	2	0.030			
	Berom	2.926	1.107	6.987	1	0.008	18.657	2.131	163.367
	Others	-35.009	5415.156	0.000	1	0.995	0.000	0.000	.
	**Marital status**								
	Married			2.586	3	0.460			
	Widow	-1.333	0.972	1.880	1	0.170	0.264	0.039	1.772
	Divorce	− 0.278	1.743	0.025	1	0.873	0.757	0.025	23.053
	Single	18.315	28420.722	0.000	1	0.999	89970898.141	0.000	.
	**Religion**	-74.629	15510.038	0.000	1	0.996	0.000	0.000	.
	**Number of sex partner**								
	1			0.000	2	1.000			
	2	-3.398	10153.823	0.000	1	1.000	0.033	0.000	.
	3 and above	10.821	10713.937	0.000	1	0.999	50054.514	0.000	.
	Family type	49.837	7083.682	0.000	1	0.994	4.404E + 21	0.000	.
	**Occupation**								
	Housewife			2.038	4	0.729			
	Civil servant	-37.653	19002.212	0.000	1	0.998	0.000	0.000	.
	Businesswoman	-39.004	19002.212	0.000	1	0.998	0.000	0.000	.
	Others	-37.822	19002.212	0.000	1	0.998	0.000	0.000	.
	Student	-17.877	23183.428	0.000	1	0.999	0.000	0.000	.
	**Annual income**								
	< 284,700.00			0.523	2	0.770			
	284,700.00479,500.00	− 0.292	1.065	0.075	1	0.784	0.747	0.093	6.020
	> 479,500	0.378	1.368	0.076	1	0.782	1.459	0.100	21.298
	Constant	67.983	39659.313	0.000	1	0.999	3.348E + 29		

Significant at p < 0.05

### Phase two (the ideation phase)

Phase two focused on the data collected through focus group discussions with four community women on the development of care-seeking behavior interventions. The opinions of an academic expert, the researcher’s supervisor, and the midwives responsible for sample collection were also sought during this phase. Six steps of thematic analysis [33] were used to analyze the qualitative

Step 1: Become familiar with the data.

Step 2: Generate initial codes.

Step 3: Search for themes.

Step 4: Review themes.

Step 5: Define themes.

Step 6: Write up.

#### First meeting

We discussed and brainstormed some interventions that may improve screening from previous studies, and these interventions include offering transport [34], having health insurance [35], offering free screening services [14], [15], [22] alternative clinic sites, having female family doctors [14], having the recommendation to attend the screening [36], a brochure, training, and verbal invitation [37].

We then asked the following questions:

Question 1: I said we may discuss this after analyzing the completed questionnaires, and you consented. What do you think we can do to improve our participation in cervical cancer screening? Remember, we are representing the opinions of the women in the community. Participant quotes:

“*We want awareness and health talks on the disease; after the health talks, screening*.” 02.

“*We will follow women to churches to create awareness and give a health talk*.” 01, 02.

“*Some said it’s only screening they want to go to*.” 04.

Question 2: How can we get the information to the whole group of women that participated in this study? Participants quoted the following:

“*Since they are all not Christians, we have Muslim participants, and some women are not in any of the women groups in their churches, we can print materials for various women church groups*.” 01.

“*They should print papers for them; they surely have someone in their family that can read*.” 02. “*Not everyone knows how to read, so I prefer women just go for screening*.” 03.

“*The problem is that getting women together is difficult, so screening is the only option*.” 04. Question 3: Screening is the only option; how can we do this screening? Participants stated,

“*JUTH is not far from here*.” 01, 02, 03:

“*Some women will still not go for the screening for transportation fare*.” 02.

“*If we bring it to Tollemach Primary School, some women who are far away will still have challenges.*” 03.

“*Even if we take the screening to the community, some women will still not go because it will be too far for them.*” 04.

“*You see, some reasons why women may not go for this screening are that some results of the samples collected will be exchanged, and they will bring out the results for others and give them to others.“ They will tell you that you have a disease even when the result does not belong to you. “People don’t want to do screening because of that*.” 02.

We have to consider those that will collect the samples for the reason you gave, which is that some results from the samples collected could be mistakenly given to others that don’t have the disease. Participants quoted the following:

“*Participants would have to go to JUTH for screening*.” 01, 02, and 03.

“*This one, women will want to go, but transportation will be their challenge*.” 01.

“*As it is rightly said, anyone with the intention will go*.” 02, 03.

“*Two hundred will not be much to transport to JUTH*.” 03.

“*We may even trek to the place.*” 01, 03, and 02.

*“We will print materials and distribute them to communities* 1, 3, and 4.

At the first meeting, we identified the following facilitating conditions:

Free screening for cervical cancer comes first.

Then, awareness should be created, and information on cervical cancer and preventive measures should be provided as screening continues. Information to improve knowledge and awareness should be provided via printed material to community women since we may not be able to gather the women together for health talks. Free transportation for participants that may not afford transport fares to the screening site.

#### Second meeting during the ideation phase

We discovered that women were reluctant to screen compared to community women’s verbal willingness to screen, and we sought an opinion from an academic expert, researcher supervisors, and oncology midwives responsible for sample collection. We then brainstormed and discussed further with previously recruited participants how to improve screening participation. Their suggestions are quoted as follows:

“*We can go through influencers such as various women leaders or other leaders who may influence women’s participation in cervical cancer screening.*” Academic expert.

*You may have to encourage the women to come for screening*” said the midwives responsible for sample collection.

“*We have to go through the women leaders of various church groups*.“ 04.

“*We have to go through peers to encourage women to screen*.” 03.

At the second meeting, we identified the following facilitating conditions:

Use of influencers such as women leaders, peers, and researchers.

What we did after the discussion was to meet with the influencers (women leaders of churches and a staff member working in a high school) within the community. Money was given to these influencers to give to willing participants for payment of the screening fees. Some participants who could not afford transport fares were transported by the researcher to the screening site.

### Phase three (the implementation phase)

In phase three, the user-centered interventions were tested for their effectiveness on women’s habits and effect on care-seeking behavior for cervical cancer screening.

Table 4 shows that all (63) participants who screened after intervention said the procedure was not embarrassing or painful, and women were not afraid.

**Table 4:** Postintervention Affect of Gwafan community women’s care-seeking behaviour (September-December 2021)

Item		Frequency (f)	Percent (%)	Mean (X)	SD
The procedure was embarrassing	Yes	0	0,0	2.00	0.000
	No	63	1000		
	**Total**	**63**	**100.0**		
The procedure was painful	Yes	0	0,0	2.00	0.000
	No	63	1000		
	**Total**	**63**	**100.0**		
I was afraid to expose my private part	Yes	0	0,0	2.00	0.000
	no	63	1000		
	**Total**	**63**	**100.0**		
I was afraid of the procedure and screening room	Yes	0	0,0	2.00	0.000
	No	63	1000		
	**Total**	**63**	**100.0**		

Table 5 shows that the screening rate increased from 11.2% preintervention to 29.7% (average mean screening score from 1.89 ± 0.316 pre-tests to 1.7000 ± 0.458 posttest), participation in screening increased from 65.8 to 89.8% between 2018 and 2021 (1.87 ± 0.343 to 1.87 ± 0.313, good experience during screening increased from 86.8 to 95.0% 1.87 ± 0.339 pre-test to 1.9500 ± 0.218 posttest).

**Table 5:** Postintervention habits of Gwafan community women’s care-seeking behaviour (September- December 2021)

Item		Pretest		Posttest		Pretest		Posttest	
		F	%	F	%	Mean	SD	Mean	SD
**I have** **ever screened**	Yes	38	11.2	101	29.7	1.89	0.316	1.70	0.458
	No	302	88.8	239	70.3				
	Total	340	100.0	340	100				
Year last screened	< 2018	13	34.2	11	10.9	1.87	0.343	1.89	0.313
	2018–2021	25	65.8	90	89.1				
	Total	38	101	100.0	100.0				
**Experience** **during** **previous screening**	Bad procedural experience	5	13.2	5	5	1.87	0.339	1.95	0.218
	Good experience	33	86.8	96	95				
	Total	38	100.0	101	100.0				

## Discussion of findings

Regarding preintervention screening habits, only 11.2% had ever screened before the interventions. Women’s age, annual income, marital status, religion, number of sexual partners, and occupation are not associated with screening. However, there is a disparity in the uptake of cervical cancer screening based on tribe. Screening areas near residences did not improve screening in line with [22], as this community is approximately 4 to 5 km from the screening site. Most women who were screened before the intervention—86.8%—had a good experience. Concerning preintervention affect, findings show that most (77.4%) women have the fear of exposing their private parts; 64.7% have the fear of screening procedures and environment in line with [21], [38], [39], [40]; 75.9% have the fear of diagnosis in line with [16], [18], [38], [39]; the majority feel the procedure is painful as found by [18], [22] [40]; and the majority feel the procedure for screening is embarrassing in line with [21], [39], [40], [41], [42], [43].

The interventions to improve screening participation based on women’s needs in this community include free screening, creating awareness on the disease and screening, offering transportation support, and the use of influencers to encourage screening as quoted: “We want awareness and health talks on the disease after the health talks, then screening.” 02. “We can go through influencers such as various women’s leaders or other leaders who may influence women’s participation in cervical cancer screening.” Academic expert “You may have to encourage the women to come for screening,“ said the midwife responsible for sample collection. “We have to go through the women leaders of various church groups.” 04. “We have to go through peers to encourage women to screen.”

The post-intervention findings indicated that screening improved from 11.2% preintervention to 29.7% (average mean screening score from 1.89 ± 0.316 pre-test to 1.7000 ± 0.458 posttest), and participation in screening increased from 65.8 to 89.8% between 2018 and 2021 (1.87 ± 0.343 to 1.87 ± 0.313) among those who were screened. This significant increase may have resulted from care-seeking behavior interventions that were based on women’s needs, such as free screening [35], [44] client reminders [34], [45], printed materials and verbal invitations [37], offering transport [34], the use of influencers such as peers [20], women leaders, and researchers, and sample collection by female midwives. Good screening experiences increased from 86.8 to 95.0% (1.87 ± 0.339 pre-test to 1.9500 ± 0.218 posttest).

More than 83% of participants who showed verbal willingness to screen during qualitative data collection and all who expressed willingness during quantitative data collection in the inspiration phase were yet to screen at the time of this evaluation, indicating that their willingness did not translate to participation, as found by [26]. This may result from fears [38], past experience, and the misconception that a woman with a negative result could be given the result of another woman whose result has cervical changes or cancer during results collection.

## Conclusion

This study was conducted to improve the care-seeking behavior of community women towards cervical cancer prevention. Screening in this community was low, resulting from fears, embarrassment, and dissatisfaction with past experiences with other screening services, even though the participants’ residences were near the screening center. Screening participation significantly increases with care-seeking behavior interventions such as free screening, sample collection by female care providers, provision of printed materials to community women, verbal invitations and reminders, and encouragement by influencers such as women leaders, peers, and researchers. Sociodemographic variables such as age, occupation, religion, income, marital status, and number of sexual partners may not predict participation in screening.

## Limitation

The study was limited to a community in Jos-North, Plateau State, North-Central Nigeria, and may not be generalized to other communities without similar characteristics. This study provides insight into care-seeking behavior and interventions that may improve screening participation. We suggest that care-seeking behavior interventions to increase participation be evaluated for six to twelve months post intervention. This study could be replicated in a wider society and in other cultures.

## Data Availability

All the data generated during this study is included in this published article.
